# Harnessing the Power of Education for a Low-carbon Future in Anaesthesia: A Narrative Review

**DOI:** 10.4274/TJAR.2026.262416

**Published:** 2026-10-30

**Authors:** Baxter Smelzer, Gabriel Chang, Shubha Srinivasareddy

**Affiliations:** 1 The Pennsylvania State University College of Medicine, Department of Anaesthesia, Pennsylvania, United States of America; 2 Penn State Health, Milton Hershey Medical Center, Department of Anaesthesia, Pennsylvania, United States of America

**Keywords:** Sustainable anaesthesia, volatile anaesthetics, carbon footprint, environmental education, low-flow anaesthesia

## Abstract

Climate change poses a significant threat to public health, with the healthcare sector being a major contributor to greenhouse gas emissions. Anaesthesia and the operating room are notable contributors within this sector, particularly because of volatile inhalation gases such as desflurane and nitrous oxide. Therefore, improving sustainability in anaesthesia is a clinical and ethical imperative. This review examines recent advances in leveraging education to promote low-carbon anaesthesia practices. We summarize evidence on how various educational interventions and formats can reduce the carbon footprint of anaesthesia and discuss implementation strategies, challenges, and future directions. Many studies demonstrate that educational programs reduce the use of high global warming potential anaesthetic agents. Programs vary widely in scope, ranging from single-session lectures and surveys to multi-year sustainability task forces and formal policy changes, some of which have resulted in up to a 90% reduction in volatile agent emissions. Combining multiple techniques (such as lectures, posters, and vaporizer labeling) has been associated with substantial reductions in volatile agent emissions and with improved organizational readiness for sustainability initiatives. These education-driven shifts toward environmentally friendly approaches, such as low-flow techniques, total intravenous anaesthesia, and regional anaesthesia, have proven effective, cutting costs, maintaining patient safety, and reducing carbon emissions. Educational initiatives represent a practical and cost‑efficient strategy to reduce carbon emissions in anaesthesia practice and in healthcare. By integrating environmental stewardship into anaesthesia training and policy, anaesthesiologists can lead the transition of healthcare to low-carbon care.

**Main Points**

• Educational interventions consistently reduce volatile anaesthetic use without policy mandates or compromised patient safety.

• Low-flow anaesthesia, total intravenous anaesthesia (TIVA), and regional techniques significantly reduce emissions, cut costs and improve operational efficiency.

• Implementation barriers such as knowledge gaps, safety misconceptions, and resource constraints are surmountable through integrated medical school curricula, evidence-based safety training, and transparent carbon tracking systems.

• Sustainable anaesthesia education requires context-specific strategies: high-income countries should promote TIVA and advanced systems, while low- and middle-income settings should prioritize cost-effective interventions such as avoiding desflurane and minimizing fresh gas flows.

## Introduction

Climate change is an ongoing concern, primarily attributable to emissions from manufacturing and automobiles. However, a major contributor on which experts have only recently focused is the healthcare system, specifically the use of volatile inhalational anaesthetics.^1^ There are two common measurements for evaluating the environmental impact of various compounds, global warming potential (GWP) and carbon dioxide equivalents (CO_2_e). GWP is an index representing the heat trapped by one kg of a compound compared to one kg of CO_2_ during a 100-year period. CO_2_e represents how many kg of CO_2_ equivalents are generated by one kg of a compound. Higher values for both GWP and CO_2_e indicate greater warming effects. Nitrous oxide, a commonly used inhalational anaesthetic, is a major contributor to ozone depletion and has a high GWP of 298.^2^ Desflurane, another common volatile anaesthetic, has a GWP of 2,540 and CO_2_e of 3,909 kg mL^-1^ which when used in one seven hour operation is equivalent to driving a car 7,850 kilometers.^3^ Another commonly used inhalation anaesthetic, sevoflurane, has a smaller but still impactful effect at 0.408 CO_2_e kg mL^-1^.^3^ Inhaled agents, which are more commonly used, tend to have a much higher CO_2_e when compared to alternative administration methods, such as total intravenous anaesthesia (TIVA), which has a CO_2_e of 0.017 kg mL^-1^.^3^ Regional anaesthesia does not have a defined CO_2_e; rather, its environmental impact primarily arises from disposable packaging and instrument sterilization processes, for which environmental measurements remain limited.

The use of volatile anaesthetics contributes substantially to the carbon footprint of operating rooms and the healthcare system. Educational interventions targeting both clinicians and administrative staff have been shown to reduce this impact.^4^ This review examines how such educational initiatives within anaesthesia departments and hospitals influence the choice of anaesthetic agents, delivery techniques, the use of volatile anaesthetics, and broader perioperative practices to reduce carbon emissions.

This study is a narrative review of educational interventions to promote environmentally sustainable anaesthesia practice. We conducted a targeted search of PubMed, Scopus, and PubMed Central for articles published between 2015 and 2025 using combinations of terms related to anaesthesia (e.g., “anaesthesia,” “perioperative”), education (e.g., “education,” “training”), and environmental impact (e.g., “carbon footprint,” “global warming potential”). A total of 221 titles and abstracts were screened for relevance to anaesthesia, educational interventions, and environmental outcomes (n=37). Included studies were from countries with significant healthcare systems and documented sustainability programs, including the United States (US), Germany, the Netherlands, the United Kingdom, Australia, and South Korea. Full-text articles were then assessed for inclusion based on relevance and methodological contribution. The final article selection was reached by consensus through discussion (n=25, see Figure 1). Reference lists of key articles were also reviewed to identify additional sources. For our core studies (see Table 1), we included twelve English-language retrospective, interventional, cohort, qualitative, and review articles that involved anaesthesia, including perioperative anaesthesia, described an educational intervention, and reported environmental outcomes. We also included foundational papers on greenhouse gas emissions, barriers, and global epidemiology to provide context and guide the discussion. In total, 25 peer-reviewed articles were included.

## Why Change is Needed?

Modern anaesthesia is safe and effective for a wide range of surgical procedures. However, its marked increase in use has revealed its negative environmental consequences. Volatile inhalation anaesthetics, such as desflurane, sevoflurane, isoflurane, and nitrous oxide, are recognized as major greenhouse gases.^4^ Their widespread use, combined with fairly low induction efficiency (95% remaining unaltered) results in massive daily releases of these greenhouse gases into the environment.^5^ This has led to anaesthesia being one of the leading sources of hospital-related emissions, with studies estimating emissions close to 5%, a disproportionately large share relative to the number of procedures that use these inhalation anaesthetics.^6, 7^

The overall impact of emissions and climate change is not confined to the environment. It plays a direct role in human health, including heat-related illnesses and respiratory issues from pollution.^8, 9^ Thus, addressing the major role of anaesthetics in CO_2_ emissions is imperative to improving the health of patients. Moreover, many of the environmentally friendly practices in anaesthesia, such as local anaesthetics and low-flow anaesthesia, align with broader administrative goals of patient safety and cost-efficiency, thereby boosting incentives to change practice.

The roadmap for implementing change is extensive. Major opportunities include switching from high-GWP to low-GWP inhalation induction agents; minimizing fresh gas flow (FGF) during inhalation anaesthesia and using low-flow or rebreathing systems; switching from inhalation induction to TIVA or regional blocks; and prioritizing reusable products. With evidence of financial incentives and clinical evidence demonstrating improved patient safety, education becomes the next step in implementing these changes in practice.

### Educational Strategies to Promote Low-carbon Anaesthesia Worldwide

Studies from across the globe describe several educational strategies that have been shown to reduce the environmental impact of anaesthetic use.

### European Educational Strategies

One effective approach involves lecture-based interventions and grand rounds within departmental teaching. In one German study, repeated lectures were delivered across three hospital campuses, after which volatile anaesthetic–related emissions reportedly dropped by 90%.^4^ These sessions emphasized the GWP of commonly used anaesthetics, particularly desflurane highlighting that its GWP is 2,540. They also proposed the use of sevoflurane and TIVA as lower-impact alternatives. Following these educational sessions, the use of desflurane in the examined hospitals declined markedly and was nearly phased out by late 2023.^4^

Building on these results, the same hospital system at Charité in Germany banned the use of desflurane through a top-down, policy-backed approach. These guideline changes, which removed vaporizer usage, updated ordering protocols, and enforced minimal-flow techniques, were all paired with reiterative sustainable anaesthetic practice education. While lecture-based education alone was effective, top-down education and policy change produced the most rapid and durable reduction in carbon output compared with the bottom-up voluntary education approach.^4^

Similar outcomes were seen in four French hospitals that created “sustainable anaesthesia groups,” or “green teams,” which were groups of anaesthesia personnel that organized information campaigns, distributed posters, and provided feedback on staff awareness and habits via online surveys. Despite participation from only 19% of staff, desflurane and sevoflurane use dropped significantly, with monthly CO_2_ emissions decreasing by about 14.16 tons following the campaign.^5^

### North American Educational Strategies

In North America, one intervention employed visual cues and behavioral nudges, including informational posters in break rooms and hallways, reference charts at work stations, and stickers on anaesthetic vaporizers.^10^ These reminders increased clinician awareness of desflurane’s GWP and encouraged lower-carbon anaesthetic choices, with providers reporting greater awareness and greater application of sustainable practices.^10^

Rather than relying solely on extracurricular groups, some departments have directly incorporated sustainability education into residency training and Continuing Medical Education programs. These efforts focused on the “5 Rs” (reduce, reuse, recycle, rethink, research) and applied circular design principles to promote a more sustainable operating room.^2^ The implementation of each component has reduced anaesthesia-related carbon emissions, and therefore integrating this circular design approach may yield greater long-term change and improve readiness for sustainable practices in anaesthesia and the broader operating-room environment.^2, 11^

Additionally, one study demonstrated the effectiveness of an audit-feedback system in which residents received weekly emails with their total FGF relative to an American Society of Anesthesia low-dose standard and to the performance of other residents. In just a four-week period, this audit-feedback program resulted in a 22% reduction in FGF, with nearly 56% of resident survey respondents indicating that the personalized data motivated a decrease in their volume usage of environmentally harmful anaesthetics.^12^ These findings suggest that timely, case-by-case reports of individual performance in anaesthetic use could contribute to reducing GWP in the operating room.

### Global/Asian Educational Strategies

In a Korean study, short-term awareness campaigns, similar to the green teams, included departmental briefings and educational handouts summarizing the environmental impact of desflurane, sevoflurane, and other techniques. These interventions led to a 50% decrease in desflurane use and significantly dropped CO_2_ emissions in the span of just one month.^5, 6^

On a global scale, professional societies have contributed to the push toward environmentally friendly healthcare practices. For instance, the World Federation of Societies of Anesthesiologists (WFSA) issued a 2022 statement urging anaesthesia programs to integrate environmental sustainability into their curricula.^13^ A global consensus among 45 anaesthesia leaders held that educating providers on sustainability is both achievable worldwide and crucial to reducing anaesthesia’s footprint. To date, the literature has not explicitly evaluated whether the WFSA 2022 statement has led to measurable changes in curricula, institutional policies, or clinical practice.

### Evidence of Impact

Recent studies have provided encouraging evidence that educational interventions are effective in reducing the carbon footprint of anaesthesia practice. One of the major effects is the sharp reduction in the use of desflurane, the inhalation anaesthetic with the highest GWP. In multiple studies, education on the negative environmental effects of desflurane, particularly through departmental programs and the creation of sustainable anaesthesia groups, led to about a 50% decrease in use.^3, 4, 5, 6^ Many of these changes were made without policy mandating bans on desflurane, because the evident effect on carbon emissions, safe and effective alternatives such as sevoflurane, and the price reduction alone provided sufficient incentive to change practice. When top-down changes were implemented through policy, the greatest decrease in desflurane use was observed.

Educational interventions also shifted induction practices away from high-flow techniques toward increased use of low-flow techniques. In particular, a German study using an educational bundle of lectures, posters, and stickers resulted in a self-reported 14% increase in low-flow anaesthetic use, with financial incentives and patient safety playing major roles in the switch.^14^ The education focused on informing providers about the advantages of low-flow anaesthesia, dispelling myths about patient safety and reduced gas use, and training providers in low-flow delivery skills. Another study quantified the effects of education by calculating carbon emissions, finding that reducing sevoflurane flow rates from 3 L min^-1^ to 1 L min^-1^ can reduce them by more than 50%.^10^ Low-flow techniques therefore illustrate the measurable impact of educational initiatives on reducing the carbon footprint of anaesthesia.

One of the largest reductions in emissions in anaesthesia practice was achieved through education on the use of TIVA and regional anaesthesia instead of inhalation anaesthetics. TIVA does not use inhalation anaesthetics, which confers a major environmental benefit compared with inhalation agents such as desflurane and sevoflurane, which do not provide efficient induction and thus produce significant environmental emissions. Furthermore, TIVA and inhalational induction have been shown to be equally effective and safe.^14, 15^ As a result, through educational programs and sustainability task forces within hospitals, there was a major increase in TIVA use and associated decrease in anaesthesia-related emissions, with one study showing a 20-fold decrease, from 48.85 kg CO_2_ to 2.42 kg CO_2_.^3^ When accounting for standard clinical dosing patterns, a single hour of desflurane anaesthesia generates approximately 179,055 grams CO_2_e vs. propofol-based TIVA at around 231 grams CO_2_e, demonstrating TIVA’s environmental benefit.^16^ Similarly, the method of anaesthetic administration has been shown to significantly affect the carbon footprint associated with its use in operating rooms. In a neurosurgical practice study that measured its carbon footprint in usage of general anaesthesia and spinal anaesthesia, the results showed a nearly 67-fold reduction from 4,745 g CO_2_ to 70 g CO_2_ in carbon footprint when switching to spinal administration from general anaesthesia. The highly significant difference in footprint measurements suggests substantial potential for footprint reduction by altering administration techniques through education.^17^

Along with the direct environmental benefits of changing anaesthesia practice, the importance of an added financial incentive should not be underestimated. Transitioning from high-cost volatile anaesthetics to lower-volume, lower-cost techniques such as TIVA and regional anaesthesia has facilitated top-down policy implementation and has resulted in substantial annual cost savings, with high-flow volatile anaesthetics (such as sevoflurane) costing approximately $7.99 per hour, compared with about $2.00 per hour for low-flow anaesthesia and TIVA.^15^ For instance, in the Netherlands, a nationwide professional guideline promoting “TIVA when possible, inhalation when necessary” helped catalyze a rapid shift in practice across multiple hospitals.^18^ What truly drove its successful adoption, however, were its strong educational outreach and framing that emphasized its cost savings and clinical safety.^18^ This combination of environmental and economic incentives provided a compelling rationale for institutional investment in sustainable anaesthesia practices, underscoring the value of integrating financial considerations into climate-conscious healthcare strategies.

Beyond quantifiable metrics such as cost or gas volume, education has had a positive effect on the mindset in anaesthesia departments and on the willingness to implement change. Most participants in educational programs reported in post-education surveys that they were more likely to prioritize environmental considerations in their work and to be open to new practices.^6^ While this cultural change is considerably more difficult to measure, it is critical to long-term sustainability efforts, as it encourages sustainability-related initiatives even after the educational programs end.

Overall, the available literature indicates that educational interventions are effective in reducing anaesthesia-related greenhouse gas emissions. Whether it is through sustainability groups, educational programs, or other methods, educating anaesthesia providers has led to measurable reductions in anaesthesia-related CO_2_ emissions, often accompanied by other ancillary benefits like reduced costs and cultural changes.

### Barriers and Challenges

Despite the effectiveness of various educational interventions to reduce the impact of volatile anaesthetics on the operating room’s carbon footprint, systemic barriers have been shown to persist at the system, provider, and institutional levels. Understanding these obstacles, along with their potential solutions, is important for creating sustainable and impactful change initiatives.

The first and most fundamental issue in implementing educational programs is that informing providers about the climate impact of anaesthetics is not part of the standard medical education curriculum. Thus, the initial encounter with an anaesthesiologist who has received formal instruction on the GWP of their anaesthetic use will take place during residency or fellowship, if at all. For instance, providers often lack awareness of desflurane’s high GWP.^4^ As a result, knowledge gaps and misconceptions persist, interfere with informed decisions regarding environmental impact, and may lead to resistance to change due to unfamiliarity.^4^ A solution in this case may be to incorporate environmental training and GWP information earlier in anaesthesia training and, preferably, into medical school curricula.^19^

Another critical misconception pertinent to efforts to reduce the environmental impact of anaesthetic use in the operating room concerns patient safety and often deters providers from such practices. The idea is that reducing fresh anaesthetic gas flow, which is a primary strategy in decreasing volatile agent emissions, leads to an increase in compound A and carbon monoxide, which are toxic to humans, thereby raising concerns about patient harm and neurotoxicity.^2^ This is a theoretical idea that there will be increased byproduct accumulation when volatile anaesthetics interact with CO_2_ absorbents. However, only excessively low flow rates may result in increased production of compound A and carbon monoxide.^2^ Modern absorbents are engineered to prevent these risks at clinically appropriate flow rates (0.5-1.0 L/min). Although it is a worthy concern for patient safety, worrying about trace byproducts from minimal-flow anaesthesia is disproportionate compared with other clinical risks. Therefore, educational solutions must be incorporated to teach updated absorbent physiology and evidence-based safety thresholds, to amend this misconception, and to verify low-flow anaesthesia as a useful practice for reducing the environmental impact of volatile agents.^2^

Similar concerns exist regarding the use of TIVA in clinical settings. TIVA has been proven to be an effective technique to reduce operating room greenhouse gas emissions, but it introduces the issue of drug waste. More than half of drugs prepared for clinical use go unused, and propofol makes up approximately 45% of this waste, which is considered environmentally unsustainable.^2^ This is because propofol is known to persist in aquatic ecosystems and to accumulate in animal tissues. Although the footprint of propofol is concerning based on available data, assessments reveal that its life cycle has a significantly lower impact than those of desflurane or nitrous oxide.^2^ Therefore, TIVA is still better than using high-GWP volatiles despite its aquatic costs, but it remains a non-zero-impact solution to the problem. Thus, it is important to provide anaesthesia personnel with comprehensive lifecycle education rather than oversimplified usage messaging.

While knowledge gaps and misconceptions continue hindering adoption of environmentally sustainable practices in the operating room, these challenges are compounded by cultural resistance and limited clinician engagement. Low voluntary participation continues to be a driving force behind the lack of adaptation and the limited adoption of greener techniques.^5^ This suggests that anaesthesia personnel and providers perceive education regarding sustainable anaesthetic use as optional rather than integral to practice. Arguably, this could be addressed with the reporting and tracking of volatile usage and of correlated GWP and environmental impact, an additional issue facing this topic. Most hospital systems lack visible tracking or targets for anaesthetic carbon footprints, thereby reducing the urgency to change practice. Reporting also lacks standardization, preventing meaningful comparisons across settings.^2^

The adoption of greener techniques also faces limitations in infrastructure and resources. TIVA, regional anaesthesia, and enhanced monitoring equipment require substantial capital investment and increased drug costs. With the US  and many other countries already facing access-to-care issues, one might expect that these techniques may not rank high on providers’ priority lists. This is a significant barrier, as the cost of implementing this change limits its adoption in resource-limited environments.^2^ Another component is the use of scavenging systems, which are not only designed to vent gases to the outside atmosphere but also, in higher-end models, incorporate technology to absorb, neutralize, or recycle these agents.^2^ Most hospitals utilize basic scavenging systems that vent rather than capture and recycle, thereby missing an opportunity to implement environmentally friendly practices. If advanced scavenging systems were made more cost-efficient, accessible, and standardized, their adoption could help reduce the environmental impact of volatile agents.

Bottom-up education and the use of organized campaigns, lectures, posters, and green teams have proven to be an effective route to reducing GWP of anaesthetic agents, but they risk regression without repeated reinforcement. When healthcare systems incorporate these techniques, they should maintain educational efforts to achieve their goal of reducing environmental impact. On the other hand, top-down policy mandates, such as desflurane vaporizer removal, will achieve a more rapid uptake within three to six months and remain durable and efficient.^3^

Many of these obstacles were echoed in a large cross-sectional study of anaesthetists in Australia and New Zealand, where, despite broad support for sustainability, practitioners reported barriers including limited infrastructure, time pressure, and lack of educational support that prevented the widespread adoption of environmentally conscious practices.^20^ Furthermore, this study found that a minority of respondents received any formal training on sustainability, highlighting the educational gap and the lack of standardization across anaesthesia curricula.

The primary clinical priorities are patient safety and positive outcomes, and in emergencies many physicians may revert to high-flow techniques and agents such as desflurane and nitrous oxide because of their familiarity and experience, thereby sidelining sustainability goals. It is important that environmental education efforts frame sustainability in alignment with clinical excellence and safety, rather than as a competitive component. Creating a balance among patient benefit, safety, cost-effectiveness, and environmental responsibility is a key component of incorporating these greener techniques.^2^

In a healthcare system’s mission to educate employees and providers about sustainable anaesthesia practices, barriers include legitimate knowledge gaps, misconceptions about techniques and lifecycle costs, cultural resistance and physician inertia, resource constraints, and competing clinical pressures. These obstacles are addressable, and several practical strategies have been proposed to overcome them. Effective educational interventions should clarify the evidence-based safety of low-flow techniques, explain the footprints and impacts of different volatile agents, demonstrate sustainability’s alignment with patient safety and economic considerations, and incorporate change and accountability into healthcare institutions.

### Special Considerations for Training and Global Equity

Because of the varying attributes of healthcare systems and settings, education in sustainable anaesthesia cannot follow a one-size-fits-all model. Settings in which anaesthetics are used and that involve different professional groups and training levels require tailored approaches that balance clinical reality, resource availability, and environmental goals. To achieve these environmental goals, it is imperative that all anaesthesiologists receive education in greening the operating room.^2^ Framing sustainability as a core clinical competence, rather than an optional ethical add-on, teaches trainers about the environmental impact of agents, alongside safety and efficacy considerations. Establishing sustainable practice as the norm will identify the overlap among cost, clinical safety, and environmental benefit.

Also, physicians who have been practicing for longer, typically attendings, hold leadership roles and have more experience. However, established providers face barriers, being entrenched in habitual practices and skeptical of new systemic changes.^21^ Education must emphasize both the environmental and economic benefits to address this cultural resistance from the top down. When institutional leaders and senior staff model sustainable practices, junior colleagues such as residents and new providers follow suit.^3^ These efforts must extend beyond physicians’ roles. Operating room staff such as Certified Registered Nurse Anesthetists (CRNAs), Certified Anesthesiologist Assistants, and other support staff play critical roles in waste management, equipment handling, and gas scavenging. Education for these staff members should focus on practical, actionable steps, such as waste segregation and reuse protocols.^2^

A major consideration for global equity is addressing differences in practice environments between high-, middle-, and low-income (LIC) countries. Countries like France and other high-income countries (HIC) have infrastructure for TIVA, advanced monitoring, reusable equipment cleaning systems, and modern absorbents for CO_2_. In these countries, education can confidently promote low flow techniques, sevoflurane minimization and regional administration without facing major resource and accessibility barriers.^4, 5, 6^ The adoption of TIVA and low-flow techniques may be hindered in resource-limited environments in LIC and middle-income countries (MIC). Healthcare systems often lack capital for TIVA pumps and infusion systems, reliable supply chains for injectable regional agents, and infrastructure for advanced waste scavenging. Rather than using TIVA and low-flow techniques as the universal solution, educational efforts should be location-specific and provide context-appropriate guidance.

Another concern regarding regional infrastructure is power-grid dependency, because carbon emissions from reusable equipment depend primarily on the local area. For instance, in countries that rely on coal-based energy, such as Australia, the use of reusable equipment may increase carbon emissions.^2^ Therefore, educational programs must be regionally calibrated to account for their power-grid composition. For example, cleaning equipment with water and increasing its reuse may actually have a negative impact on climate change if conducted in an area that does not have much water to begin with.^2^ Local accessibility and resources are therefore key components in evaluating the effectiveness of sustainability efforts.

Importantly, sustainability is not typically achieved by a single entity but rather through the collective efforts of multiple groups. All stakeholders must act to ensure an effective response, including hospitals, medical and nursing societies, and state, local, and federal governments. It is important that the HICs with resources pioneer sustainable anaesthetic practices and share knowledge, equipment, and financial support with LICs and MICs. HICs and their prominent healthcare systems should develop and disseminate contextualized educational toolkits for LICs and MICs, and advocate for technology transfer and equipment donation programs. Lobbying for policy development, such as supporting desflurane bans, would also further sustainability efforts in LICs and MICs. Wealthier institutions can model and subsidize sustainability investments, thereby creating pathways for others to follow.^2^

Effective sustainable anaesthesia education must be tailored to individual operating room environments to account for substantial differences in infrastructure and resources. HICs can confidently promote TIVA and reusable equipment, while LICs and MICs benefit most from high-impact, low-cost interventions (avoiding desflurane and using minimal flows). Implementation must balance safety, cost-effectiveness, resource availability, and environmental responsibility specific to each location.^2^

### Education and Waste Management

While this review focuses primarily on the use of education in anaesthetic choice and technique, waste management is a complementary domain in which education can further reduce anaesthesia’s environmental footprint. Educational efforts should therefore target these three high-impact areas: reusable vs. single-use equipment, drug waste reduction, and waste segregation practices.

Life-cycle assessments consistently demonstrate that reusable devices generate lower greenhouse gas emissions and less solid waste per use compared with their single-use equivalents, while also reducing long-term costs.^22^ Therefore, implementing the aforementioned educational initiatives that emphasize safety, environmental benefits, and proper use of reusable instruments may be an effective strategy for reducing anaesthetic-related carbon emissions.

Over-preparation of propofol and other agents and improper disposal also contribute to increased anaesthetic-related environmental impact; a survey of 118 anaesthesiologists and CRNAs showed that many practitioners disposed of unused anaesthetic agents down drains and into trash bins.^23^ These improperly disposed compounds may lead to downstream effects, including aquatic toxicity and other negative environmental impacts.^23^ Thus, developing educational interventions that encourage the use of smaller vials, tailoring preparation to case-specific needs, and promoting proper waste segregation may help reduce drug waste and, consequently, environmental impact.^23^ Overall, limited evidence exists regarding the impact of educational interventions on anaesthesia-related waste management strategies, indicating a need for further research in this field.

### Future Directions

While substantial progress has been made in achieving low-carbon anaesthetic practice, there are still many questions for future research. A major example is the determination of the long-term efficacy of these educational interventions. Do the decrease in desflurane use and the increase in TIVA last over 5-10 years after the educational program ends? Because many of these interventions have occurred within the last few years, there have been no major studies addressing this question. Follow-up studies and surveys could explore the lasting impact of educational interventions, as well as show the potential benefits of “booster” educations to reinforce sustainable practice.

Another direction for implementing sustainable practice is to standardize sustainability education. This could take several forms, such as developing a sustainability curriculum within anaesthesia departments or residency programs, which could include interactive exercises or lunch lectures. Additionally, including anaesthesia-related environmental impact in professional exams could improve providers’ awareness of these issues. Stemming from this idea, a multi-specialty initiative has advocated for embedding sustainability into perioperative medical education and board certification, recognizing that formal training is key to empowering clinicians to lead environmental change.^24^ However, resistance is likely, given the already-packed training schedules of anaesthesia providers.

Technological innovations also offer a promising means of reducing the carbon footprint, largely without changing anaesthesia practice. A major example is the improvement of anaesthesia gas capture and recycling systems. This would reduce the total amount of anaesthesia purchased and used, thereby providing a financial incentive for hospital systems to invest in their development. In particular, technologies that capture and reprocess waste sevoflurane have shown early promise, with experimental systems demonstrating the ability to reclaim a significant proportion of the exhaled anaesthetic for reuse.^25^ These approaches could mitigate the environmental impact while maintaining clinician familiarity and workflow. Additionally, developing novel induction methods or alternative synthetic agents could greatly reduce the environmental impact and the cost of anaesthesia. Xenon, for example, is an excellent anaesthetic and has no negative environmental impact.^15^ However, its high cost and limited availability have restricted its use. Research into its recycling and production could be an effective approach to reducing carbon emissions while supporting the provision of care.

While top-down measures have the greatest impact on changes in practice, the impact of a bottom-up approach, beginning with advocacy by anaesthesiologists, should not be underestimated. Equipping anaesthesiologists with the knowledge and confidence to engage in healthcare policy could amplify the demand for sustainable anaesthesia practices and ultimately lead to change.

Finally, addressing which anaesthesia strategy is safe and provides optimal patient care is vital to justify changes to current practice. If an alternative induction agent or method is more environmentally friendly but carries a greater risk of complications during surgery, no provider will be willing to risk patient harm or their license by changing their anaesthetic practice. On the other hand, if there is clinical evidence that an alternative induction agent or method is safer than the current standard, practicing anaesthesiologists and hospital administrators will be much more likely to adopt the practice. Thus, research to understand the specific clinical implications of alternative induction agents can be crucial to adopting environmentally friendly anaesthesia practices.

Exploring these future directions makes it clear that the quest for sustainable anaesthesia remains an ongoing endeavor. Pushback can be resolved only through additional research and evidence, which will, in turn, facilitate education and implementation. The anaesthesia field has been cognizant of the need for change toward sustainable anaesthesia, and many changes have already been institutionalized.^3^ By staying informed and proactive, the field of anaesthesia will remain at the forefront of both medical advances and environmental responsibility.

## Conclusion

Anaesthesiology stands at an inflection point where clinical excellence is becoming inseparable from environmental stewardship. This review demonstrates that education is a practical and scalable method for transforming anaesthesia practice toward low-carbon care. Through deliberate educational interventions, ranging from residency training in low-flow techniques to multi-year departmental sustainability campaigns and audit-and-feedback systems, anaesthesia departments have consistently achieved substantial reductions in greenhouse gas emissions from volatile anaesthetic agents. Further, these reductions have been accomplished without sacrificing patient safety or clinical outcomes, and many of these low-carbon practices align with improved cost-effectiveness and operational efficiency.

For anaesthesiologists, the implications are both clear and actionable. Incremental changes in daily practice—avoiding high-GWP agents, minimizing FGFs, optimizing technique selection, and reducing pharmaceutical waste—result in measurable environmental gains. Departmental leadership must prioritize sustainability education within residency and quality improvement programs. By integrating environmental stewardship into training, measurement systems, and institutional policies, anaesthesiologists can lead healthcare’s transition to low-carbon care while maintaining clinical excellence. The evidence is now compelling enough that sustainable anaesthesia should be treated as a quality improvement standard, ensuring we care for patients today while safeguarding the world tomorrow.

## Figures and Tables

**Figure 1:**
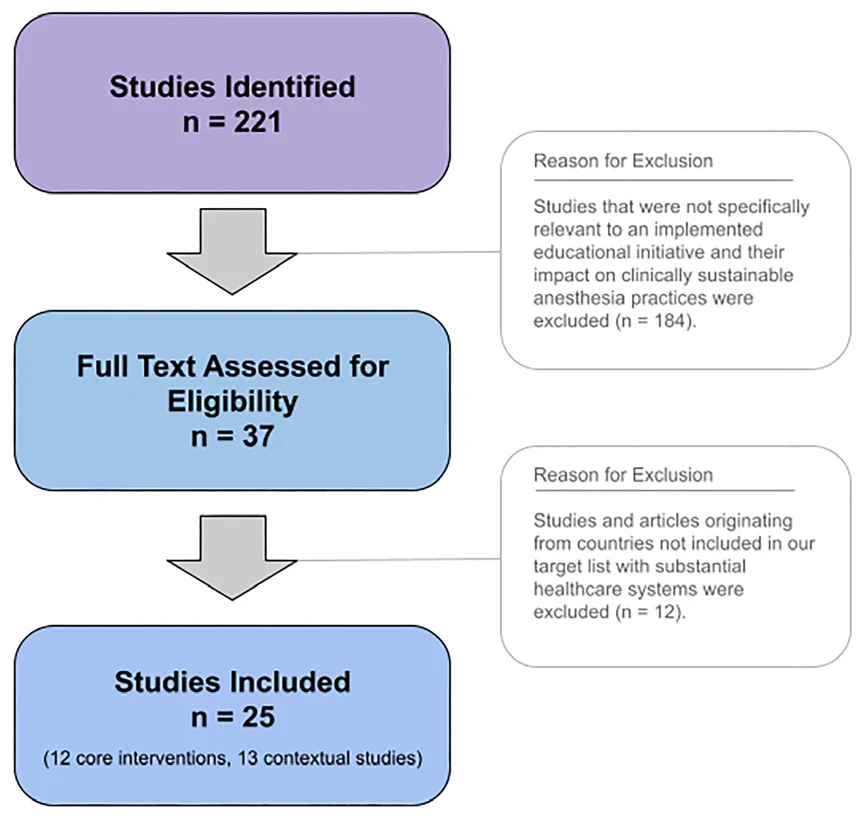
Selection criteria. Outline of the process for excluding and selecting studies to be included in the narrative review.

**Table 1. Summarized Core References:** 

**Citation (authors, reference number, year)**	**Study type**	**Main focus**	**Key findings related to carbon footprint**
Saadat and Raman^2^, 2024	Narrative review	Conceptual framework for “sustainable anaesthesia”	Identifies desflurane and N_2_O as major high‑GWP contributors; outlines low‑flow, TIVA/regional techniques, and equipment/waste changes as practical ways to reduce anaesthesia’s climate impact while maintaining safety.
Bernat et al.^3^ , 2024	Large retrospective cohort	TIVA vs. volatile‑based general anaesthesia	Demonstrates ≈20‑fold lower CO_2_e per case with TIVA (≈2.4 vs. 48.9 kg CO_2_e) compared to a mixed volatile strategy, with similar clinical outcomes, supporting TIVA as a major mitigation option.
Schwiethal et al.^4^ , 2025	Before-after interventional study	Flagship education + policy example (Charité)	Repeated education with top‑down desflurane removal achieved ≈90% reduction in volatile‑related CO_2_e and substantial drug cost savings without compromising clinical care.
Chambrin et al.^5^, 2023	Retrospective multicenter cohort	“Green teams” and information campaigns	Formation of sustainable anaesthesia groups and campaigns led to significant drops in desflurane/sevoflurane use and a steeper decline in halogenated anaesthetic CO_2_e (≈-14.16 tons/month).
Park et al.^6^, 2024	Single‑center retrospective study	Short‑term focused education	One‑month education on climate impact of inhaled agents reduced desflurane use by ~50%, increased sevoflurane, and significantly lowered OR‑level anaesthetic CO_2_e and costs.
Zuegge et al.^10^, 2019	Before-after interventional study	Visual cues + education (vaporizer labeling)	Provider education and vaporizer labels reduced purchasing of high‑GWP volatiles, resulting in decreased anaesthetic‑gas-related CO_2_e and drug expenditures.
MacNeill et al.^11^, 2024	Narrative review	Perioperative systems context	Positions volatile anaesthetics as high‑impact nodes within the perioperative carbon footprint and recommends low‑flow, desflurane avoidance, TIVA, and regional techniques to reduce emissions.
Nordin et al.^12^ , 2024	Prospective educational intervention	Audit‑and‑feedba ck program for residents	Personalized FGF feedback emails to residents reduced average FGF by ~22% in four weeks, implying a meaningful decline in waste anaesthetic gas emissions during training.
White et al.^13^ , 2022	Global consensus statement	Professional endorsement of sustainability	WFSA consensus identifies volatile agents and N_2_O as key GHG sources and calls for education and practice changes (low‑flow, TIVA/regional, desflurane/N_2_O avoidance) to reduce anaesthesia’s carbon footprint worldwide.
Baumann et al.^14^, 2025	Cross‑sectional survey	Readiness for climate mitigation	Anaesthesiologists widely recognize the need to mitigate anaesthesia ‑related emissions but report inconsistent adoption of low‑flow and desflurane‑avoidance behaviors, highlighting an attitude-behavior gap.
Wang et al.^17^ , 2022	Comparative observational study	Regional vs general anaesthesia	In single‑level TLIF surgery, spinal anaesthesia produced ≈67‑fold lower anaesthetic‑related CO_2_e (70 g vs. 4,745 g) than general anaesthesia, illustrating the footprint impact of technique selection.
Kampman et al.^18^, 2025	National implementation study	Dutch “decarbonising anaesthesia” program	Shows that a national “TIVA when possible, inhalation when necessary” strategy with education and guidelines reduced volatile gas use and associated emissions across Dutch hospitals.

TIVA, total intravenous anaesthesia; GWP, global warming potential; CO_2_e, carbon dioxide equivalents; FGF, fresh gas flow
